# Infection and colonization by *Stenotrophomonas maltophilia*: antimicrobial susceptibility and clinical background of strains isolated at a tertiary care centre in Hungary

**DOI:** 10.1186/s12941-014-0058-9

**Published:** 2014-12-31

**Authors:** Emese Juhász, Gergely Krizsán, György Lengyel, Gábor Grósz, Júlia Pongrácz, Katalin Kristóf

**Affiliations:** Diagnostic Laboratory of Clinical Microbiology, Institute of Laboratory Medicine, Semmelweis University, Budapest, Hungary; Institute of Medical Microbiology, Semmelweis University, Budapest, Hungary; Hungarian Defence Forces, Military Medical Centre, Budapest, Hungary

**Keywords:** *Stenotrophomonas maltophilia*, Antibiotic susceptibility

## Abstract

**Background:**

*Stenotrophomonas maltophilia* is an important opportunistic, mainly nosocomial pathogen that emerged in the last decades worldwide. Due to its inherent extended antibiotic resistance, therapeutic options are strongly limited. New resistance mechanisms in *S. maltophilia* make antibiotic therapy even more difficult. The aim of our study was to investigate the antimicrobial resistance of *S. maltophilia* isolates collected in our laboratory and to reveal related clinical background.

**Method:**

Consecutive non-duplicate *S. maltophilia* isolates (n = 160) were collected in a three-year period. Conventional methods, automated identification system and MALDI-TOF MS was used for identification, ERIC-PCR for genetic relationship analysis and broth microdilution method to determine the susceptibility for trimethoprim/sulfamethoxazole (SXT), ciprofloxacin, levofloxacin, moxifloxacin, colistin, doxycycline and tigecycline. Clinical final reports were used retrospectively to collect clinical information.

**Results:**

ERIC-PCR revealed large heterogeneity. Trimethoprim/sulfamethoxazole, moxifloxacin and levofloxacin were found to be the most effective agents with MIC50/MIC90 0.5/1, 0.25/1, 1/2 mg/l, respectively. Seventy percent of patients with *S. maltophilia* infection were treated in intensive care units. All-cause mortality rate was 45%. Nearly 70% of the isolates were collected from polymicrobial infections/colonizations.

**Conclusions:**

Trimethoprim/sulfamethoxazole is the most potent antibiotic agent against *S. maltophilia*. In case of SXT hypersensitivity, intolerance or resistance, fluoroquinolones are alternative therapeutic options. Missing clinical breakpoints, consensus antibiotic susceptibility testing guidelines and clinical trials make the interpretation of antibiotic susceptibility testing results difficult. The indirect pathogenicity of *S. maltophilia* in polymicrobial infections or colonizations has to be taken into consideration.

## Background

*Stenotrophomonas maltophilia* has emerged as an important opportunistic and nosocomial pathogen in recent years worldwide [1,2]. Behind *Pseudomonas aeruginosa* and *Acinetobacter baumannii*, *S. maltophilia* is the third most common non-fermenting Gram-negative bacillus responsible for healthcare-associated infections [1]. Community-acquired *S. maltophilia* infections have also been reported [3]. Pneumonia and bacteraemia are the most frequent infections [1,2]. Due to its inherent extended antibiotic resistance, therapeutic options are strongly limited [1,2,4]. Currently only trimethoprim/sulfamethoxazole (SXT) is recommended for therapy, but some circumstances (hypersensitivity of the patient, resistance of the bacterium) can limit the use of this drug [1,2]. In such cases, susceptibility of *S. maltophilia* isolates for other antimicrobials must be tested, even if clinical evidences for their efficacy are lacking yet [4]. Increasing number of patients at risk (immunocompromised patients, cancer patients, patients undergoing long-term intensive care, etc.), the necessity of the usage of broad-spectrum antibiotics such as carbapenems (which cause selective pressure for the inherently carbapenem-resistant *S. maltophilia*) and the natural features of this bacterium (biofilm forming ability, colonizing moist hospital environments) together mean that *S. maltophilia* is a continuous threat we have to face [1,2,5]. The high attributable mortality rates and poor outcomes reported in *S. maltophilia* infection makes the spread of this bacterium even more worrisome [6]. The emergence of new resistance mechanisms in *S. maltophilia* requires substantial monitoring and reporting of antibiotic suspectibility of clinical isolates [5].

The aim of our study was to investigate the antimicrobial resistance of *S. maltophilia* isolates collected in Diagnostic Laboratory of Clinical Microbiology, Institute of Laboratory Medicine, Semmelweis University (Budapest, Hungary) and to reveal whether isolates were infective or colonizers, the type of infections, the predisposing factors of infected patients, antibiotic therapy and the outcome of infections.

## Materials and methods

A total of 160 consecutive non-duplicate *S. maltophilia* isolates from a three-year collection period (2009–2011) were investigated in our study. The identification of the isolates was performed with conventional methods, VITEK 2 Gram-negative identification cards (bioMérieux, Marcy l’Etoile) and additionally by MALDI-TOF mass spectrometry (Bruker Daltonics, Bremen). For MALDI-TOF MS identification, the direct smear and 1 μL alpha-Cyano-4-hydroxycinnamic acid matrix overlay method was applied [7]. Parameters of the device were set according to the recommendation of Bruker. Identifications were assigned using the Bruker Biotyper 2.0 software. Biotyper score ≥2.0 were accepted as valid species level identification. Identification results as “*Stenotrophomonas maltophilia”*, “*Stenotrophomonas maltophilia* (*Pseudomonas beteli*)”, “*Stenotrophomonas maltophilia* (*Pseudomonas hibiscicola*)” were all accepted. *Pseudomonas hibiscicola and P. beteli* are belonging to Stenotrophomonas rRNA lineage [8].

The isolates were divided into two groups: isolates of patients who were infected by *S. maltophilia* and of those who were colonized only. Infection or colonization was distinguished according to clinical diagnoses given in final reports. Selected cases were discussed with physician to reveal clinical relevance of isolates. The definitive diagnosis of infection was clinically established. Colonization was defined as the presence of *S. maltophilia* on skin, mucous membranes, in wounds, or in excretions or secretions without causing adverse clinical signs or symptoms. Isolates of infected patients (n = 100) were cultured from blood (n = 25), bronchoalveolar lavage sample (n = 30), tracheal aspirate (n = 31), sputum (n = 7), central venous catheter (n = 4), peritoneal fluid (n = 3). Isolates of colonized patients (n = 60) were cultured from rectal swab (n = 11), urine (n = 8), ear swab (n = 6), throat - (n = 3), nose - (n = 4), eye swab (n = 7), catheter (n = 1), sputum (n = 7), tracheal aspirate (n = 7) and wound sample (n = 6).

The minimal inhibitory concentrations (MICs) of seven antibiotics were determined by the reference broth microdilution method in cation-adjusted Mueller-Hinton broth [9]. MIC values of the invasive isolates were tested by agar dilution method and by gradient diffusion test, too [9-11]. The antibiotics tested included SXT (0.25-128 mg/L), ciprofloxacin (0.5-256 mg/L), moxifloxacin (0.064-32 mg/L), levofloxacin (0.064-32 mg/L), colistin (0.25-256 mg/L), doxycycline (0.064-32 mg/L) and tigecycline (0.064-32 mg/L). The European Committee on Antimicrobial Susceptibility Testing (EUCAST) has *S. maltophilia* specific clinical breakpoint only for SXT [12]*.* Therefore, non-species related breakpoints of EUCAST were applied for fluoroquinolons and tigecycline. For doxycyline – due to absence of non-species related breakpoints – the epidemiological cut-off (ECOFF) value of *S. maltophilia* (8 mg/L) was applied. For colistin – lacking non-species related break-points and approved ECOFF - *Pseudomonas* sp. specific breakpoint (4 mg/L) was used. *Escherichia coli* ATCC 25922, *Pseudomonas aeruginosa* ATCC 27853 and *Staphylococcus aureus* ATCC 29213 were used as quality control strains.

Enterobacterial Repetitive Intergenic Consensus PCR (ERIC-PCR) was used for molecular typing of isolates, as described by Silbert *et al.* [13]. Isolates from the same ward were tested within the same PCR amplification and gel electrophoreses run. Band patterns obtained by ERIC-PCR were visually evaluated in the absence of appropriate software. Isolates with two or more different bands were interpreted as unrelated.

Clinical data and laboratory findings (white blood cell number (WBC), C-reactive protein (CRP) and procalcitonin (PCT)) of patients at the time of *S. maltophilia* infections were collected retrospectively from clinical final reports. Details of antibiotic therapy used in *S. maltophilia* infections were investigated.

Patient characteristics were tested for their association with overall mortality. First, univariate analysis using chi-squere test or Fisher exact test was performed. A p-value of <0.05 was considered as significant. Variables with significant association with mortality in the univariate analysis were entered in a multivariate forward stepwise logistic regression model to identify independent risk factors for death. The odds ratio (OR) with the corresponding 95% confidence interval (95% CI) for each variables were calculated. A p-value of <0.05 was considered indicative of statistical significance. Stata 12 software (StataCorp LP, USA) was used for the statistical analysis.

## Results

Biotyper 2.0 softver of Bruker MALDI-TOF MS classified 77% of the isolates as *S. maltophilia*, 16% as *S. maltophilia* (*P. hibiscicola)* and 7% as *S. maltophilia* (*P. beteli)*.

ERIC-PCR resulted in highly diverse patterns, however seven identical patterns among colonizer (4 times 2 isolates and 3 times 3 isolates with the same band patterns) and twelve among infective isolates (6 times 2 isolates, 4 times 3 isolates and once 5 isolates and once 6 isolates with the same band patterns) were found. In order to prevent the distortion of values by clonal isolates, 10 colonizer and 23 infective isolates were excluded from the final evaluation of antibiotic susceptibility testing results.

The results obtained with microdilution, gradient diffusion and agar dilution methods were in concordance. However, results obtained with agar dilution - especially in case of SXT - were difficult to evaluate due to slight growth of microcolonies at adjacent antibiotic concentrations. The MIC50 and MIC90 values, MIC ranges and susceptibility in % of selected isolates with different ERIC-PCR patterns were summarized in Table 1. However, MIC values did not differ significantly if the total of 100 infective and 60 colonizer isolates were evaluated.

**Table 1:** **Summary of MIC values and interpretations**

Antimicrobial agent breakpoints (mg/L)*	**Isolates**	**MIC (mg/L)**			**% of isolates**		
		**MIC range**	**MIC 50**	**MIC 90**	**Resistant**	**Intermediate**	**Susceptible**
SXT S ≤ 4, R > 4	infective	<0.25- > 32	0.25	1	1	0	99
	colonizer	<0.25- > 32	0.5	1	2	0	98
ciprofloxacin S ≤ 0.5 , R > 1	infective	<0.5-64	2	8	54	22	24
	colonizer	0.5-128	2	8	76	12	12
levofloxacin S ≤ 1 , R > 2	infective	0.125-16	1	2	7	18	75
	colonizer	<0.064-4	0.5	2	4	12	84
moxifloxacin S ≤ 0.5 , R > 1	infective	<0.064-8	0.25	1	7	6	87
	colonizer	<0.064-4	0.125	0.5	4	6	90
doxycycline**	infective	0.125-4	1	2	Insufficient evidence		
	colonizer	0.125-4	1	2			
tigecycline S ≤ 0.25, R > 0.5	infective	0.125-16	0.5	2	50	38	12
	colonizer	0.125-16	0.5	2	14	51	35
colistin S ≤ 4, R > 4	infective	1- > 256	64	>256	91	0	9
	colonizer	0.25- > 128	16	>128	77	0	23

Number of infective isolates = 77

Number of colonizer isolates = 50

*Breakpoints according to EUCAST. TMP-SMX breakpoints are specific for *S. maltophilia.* Fuoroquinolone and tigecycline breakpoints are non-species related. Colistin breakpoints are *Pseudomonas* sp. specific.

**ECOFF of doxycycline of *S. maltophilia* is 8 mg/l.

The susceptibility rates of infective and colonizer *S. maltophilia* isolates were compared. Both groups showed high rate of susceptibility to SXT. Apart from ciprofloxacin, the infective isolates had higher rates of non-susceptibility than had colonizers. Non-susceptibility rate for colistin and tigecycline were 1.2-1.3 times higher in the infective group.

The analysis of clinical background of 100 infective isolates revealed that 70% were cultured from patients admitted to intensive care units (ICU). Sixty-two isolates were obtained from patients with pneumonia (12 of them developing respiratory failure). Forty-six patients had sepsis (23 of them developing severe sepsis, septic shock or multiorgan failure), in 19 cases *S. maltophilia* was considered as the ethiological agent. The co-morbidity was chronic obstructive pulmonary disease (COPD) in 19 cases and malignancy in 19 cases, respectively. Nine patients were immunsuppressed (3 of them had lung transplantation). Clinical data and their correlation with mortality are shown in Table 2. The all-cause mortality was 45%. In 25% of fatal cases *S. maltophilia* was regarded to have direct role in death. Twenty patients have not received specific antimicrobial therapy for *S. maltophilia* (13 of them died); however, patients received antibiotics against co-infective bacteria. In 11 cases colistin was applied, 9 of them were fatal. Twenty-nine patients were treated with SXT, 7 of them died. Six patients were treated with ciprofloxacin (5 died), 17 with moxifloxacin (3 died), 16 with levofloxacin (7 died), one with tigecycline (1 died).

**Table 2:** **Univariate analysis of overall mortality of 100 patients infected by** ***S. maltophilia***

	**Died (n = 45)**	**Survived (n = 55)**	**p value**
	**No (%)**	**No (%)**	
Age in years, median (range)	67 (0–88)	62 (0–88)	-
Gender, male	23 (51.1)	35 (63.6)	0.29
Hematological malignancy	3 (6.6)	3 (5.4)	0.31
Advanced cancer	10 (22.2)	8 (14.5)	0.54
Diabetes mellitus	16 (35.5)	16 (29.1)	0.22
Corticosteroid use	4 (8.8)	8 (14.5)	0.31
Chemotherapy	4 (8.8)	11 (20)	0.2
Neutropenia (<0.5 G/L)	5 (11.1)	2 (3.6)	0.24
Post-transplantation stage	0	5 (9.1)	-
Chronic heart disease	15 (33.3)	18 (32.7)	0.88
Chronic kidney disease, hemodialysis	10 (22.2)	5 (9.1)	0.12
Chronic lung disease	13 (28.8)	14 (25.4)	0.87
Chronic liver disease	5 (11.1)	7 (12.7)	0.95
Hypertension	30 (66.6)	25 (45.4)	0.05
Admission to intensive care unit	43 (95.5)	32 (58.2)	**0.00005**
Need for vasopressors	26 (57.7)	8 (14.5)	**0.00001**
Central venous catheter	42 (93.3)	29 (52.7)	**0.00001**
Need for mechanical ventilation	41 (91.1)	28 (50.9)	**0.00004**
Severe sepsis, septic shock, multiorgan failure	23 (51.1)	5 (9.1)	**0.00001**
Non-*S. maltophilia* bloodstream infection	15 (33.3)	9 (16.3)	0.08
*S. maltophilia* bloodstream infection	12 (26.6)	13 (23.6)	0.9
Recent surgery	18 (40)	12 (21.8)	0.08
Polymicrobial infection	35 (77.7)	33 (60)	0.09

p-value < 0.05 was considered to be significant.

Data in boldface are significant.

The mortality was significantly associated with the following variables: ICU admission, need for mechanical ventilation, vasopressor therapy, presence of multiorgan failure and central venous catheter. The association with mortality also remained significant after their adjustment for age and gender. Multivariate analysis with forward stepwise logistic regression identified vasopressor therapy (OR: 0.23, 95% CI: 0.08-0.65, p = 0.006) and central venous catheter (OR: 0.15, 95% CI: 0.03-0.59, p = 0.007) as independent determinants of mortality.

The count of WBC of infected patients ranged 0.05-37.7 Giga/L, median value was 11.2 Giga/L. Values of CRP and PCT ranged 0.4-423 mg/L and 0.15-100 ng/mL, median values were 86 mg/L and 1.6 ng/mL, respectively.

Other microorganisms were isolated together with *S. maltophilia* from 68% of specimens. Numbers of these isolates are shown in Table 3.

**Table 3:** **Other microorganisms isolated together with 160** ***S. maltophilia*** **isolates**

	**Infective (n = 100)**	**Colonizer (n = 60)**
	**n**	**n**
**Gram negative**		
*Acinetobacter baumannii*	7	1
*Pseudomonas aeruginosa*	24	7
other non-fermenting*	0	3
Enterobacteriaceae**	17	13
**Gram positive**		
Coagulase negative *Staphylococci*	4	9
*Staphylococcus aureus*	8	7
*Enterococcus sp.*	10	8
**Candida sp.**	36	6

*Alcaligenes faecalis, Achromobacter xylosoxidans, Pseudomonas fluorescens **Proteus mirabilis, Serratia marcescens, Escherichia coli, Klebsiella pneumoniae, Klebsiella oxytoca, Enterobacter aerogenes, Enterobacter cloacae.

n: number of isolates.

## Discussion

Our study evaluated the antibiotic susceptibility of 160 *S. maltophilia* isolates. In accordance with international data, SXT was found to be the most effective antimicrobial agent, so it is still the first recommended agent for infections caused by *S. maltophilia*. Although resistance rates are increasing, in our study only four SXT non-susceptible isolates (2.5%) were detected. This low resistance rate is in concordance with European and North-American data (2-10%) [1,2,14,15].

Fluoroquinolones represent alternative treatment options of *S. maltophilia* infections. It was shown that fluoroquinolone and SXT monotherapy can achive equal efficacy [16,17]. Unlike ciprofloxacin for which non-susceptibility was found to be high, levofloxacin and moxifloxacin were highly effective against the tested isolates *in vitro*. Low MIC values of moxifloxacin should be emphasized. This is in concordance with many other studies [1,5,9,15]. Levofloxacin and moxifloxacin have the additional advantage of disrupting *S. maltophilia* biofilms and reducing biofilm mass, even in subinhibitory concentrations [1,2,18]. Moreover, applying levofloxacin as inhalation therapy in respiratory tract infections, the achievable concentration (50–100 mg/L) is much higher than the highest MIC value (16 mg/L) in our study. These effects increase the clinical value of levofloxacin, especially in respiratory tract infections. It is worth noting that the emergence of resistance to fluoroquinolones has been observed in *S. maltophilia* [19]. The rate of developing resistance during monotherapy was found 30% for fluoroquinolones and 20% for SXT in a previous study [16]. Hence combination therapy has been recommended in order to avoid emergence of resistance and to provide synergism between antimicrobial agents [1,6,16,20]. In the absence of clinical trials it is still an ongoing debate whether treatment with combination of antibiotics is superior to monotherapy. Combination therapy can be suggested in severe invasive infections and for immunocompromised patients until further clinical evidences are available [6].

Susceptibility for doxycycline was investigated instead of the often tested minocycline, since the latter is commercially not available in Hungary. Using *S. maltophilia* specific ECOFF, all the isolates appeared to be sensitive to doxycyline. Since ECOFF and well-established clinical breakpoints can differ significantly, it has to be assessed wether this agent is a reliable therapeutic choice. I*n vitro* activity of tigecycline against *S. maltophilia* was determined as MIC50/MIC90 0.5/2 mg/L, which is in concordance with international data [15]. Using EUCAST non-specific breakpoints, 65% and 88% of our colonizer and infective isolates were non-susceptible to tigecycline, respectively. However, using EUCAST breakpoints established for *Enterobacteriaceae* (S ≤ 1 mg/L, R > 2 mg/L) only 14% and 18%, while with breakpoints established by USFDA for *Enterobacteriaceae* (S ≤ 2 mg/L, R > 8 mg/L) only 10% and 4% of the isolates were non-susceptible, respectively. Due to uncertain interpretation the role of tigecycline as alternative choice in the treatment of *S. maltophilia* infections is unclear. Clinical efficacy of tigecycline in *S. maltophilia* infections should be investigated. However, its synergism with SXT and colistin in combination therapy was reported [15,21].

Colistin was found to have weak *in vitro* activity against our isolates, regardless of the testing method. Different studies showed resistance rates of *S. maltophilia* to be 7-100% for colistin, depending on the testing methods and the breakpoints used [1,22]. Our results show that colistin cannot be used in monotherapy in *S. maltophilia* infections; however it can show synergism with certain agents [21].

Apart from SXT, it is still not decided which testing method to use for different antimicrobial agents. EUCAST declared that antibiotic susceptibility testing of *S. maltophilia* is difficult since results are significantly influenced by several conditions including incubation temperature, culture medium or technique [4]. Susceptibility testing of SXT and doxycyline however was proved to be method independent and more reproducible than that of other agents [11]. Apart from these two antibiotics, interpretation is recommended after prolonged, 48 h incubation [11]. In our study MIC values determined after 20 hours of incubation were accepted, while CLSI method guideline was applied. Furthermore, susceptibility testing methods and breakpoints might be different depending on the site of infection: *S. maltophilia* isolates from the respiratory tract of patients with CF or other chronic lung diseases and isolates from bloodstream infections should be tested and interpreted in a different way [5]. Further studies are required to clarify these questions.

The majority of *S. maltophilia* strains were isolated from patients with polymicrobial infection or colonization. Interspecies interactions have an important role in bacterial virulence: *S. maltophilia* can protect other bacteria from antibiotics by degrading antimicrobial agents [23]. The indirect pathogenicity of *S. maltophilia* due to its β-lactamases was demonstrated [23]. While *P. aeruginosa* was the most frequent co-pathogen in our study, it should be considered that piperacillin-tazobactam, cefepime and carbapenems can be useless anti-Pseudomonas agents in an infection, where *S. maltophilia* expressing L1 and L2 β-lactamases is also present. *Stenotrophomonas maltophilia* as a co-colonizer might have a detrimental impact on *P. aeruginosa* infections treated with aminoglycosides [6]. Quorum-sensing molecules produced by *S. maltophilia* can also influence co-pathogen or co-colonizer bacteria [1,24]. Moreover, the presence of *S. maltophilia* in a polymicrobial community might lead to emergence of antibiotic resistance, since this species is carrying resistance plasmids or transposons that facilitate the spread of resistance integrons to other bacterial species [1]. Species belonging to *Enterobacteriaceae* family were the second most frequent co-pathogens in this study, including even multidrug resistant, VIM-4 carbapenemase producing *Enterobacter cloacae* strains [25]. Whether *S. maltophilia* affected the resistance pattern of *E. cloacae* strains or *vica versa,* is a further complex question. It is yet to be clarified whether drug resistance determinants are transmitted between *S. maltophilia* and other bacteria within biofilms [5]. Other microorganisms causing infection together with *S. maltophilia* can influence the clinical outcome as well [16].

Based on partial *gyr*B gene sequences, *S. maltophilia* complex can be divided into distinct groups which differ in antimicrobial resistance rates [26]. Further investigations are required to clarify whether there is a relation between the protein mass spectra of *S. maltophilia* group and antibiotic resistance.

Most infections caused by *S. maltophilia* were associated with severe morbidity and long-term, extensive ICU treatment. Previously published mortality rates vary between 14-62% [16]. The high all-cause mortality rate (45%) we observed can be connected to the serious underlying illnesses rather to *S. maltophilia* itself. However, the strict attributable mortality rate (11%) was also high, therefore clinical significance of *S. maltophilia* infections must be considered particularly in patients admitted to ICUs [27]. The fact that nearly 70% of infective isolates were collected from lower respiratory tract samples has to be considered as a limitation of our study.

## Conclusions

*S. maltophilia* is still a challenging multiresistant nosocomial pathogen. Our results show that SXT is the most potent antibiotic drug against *S. maltophilia*. Due to the low frequency of moxifloxacin and levofloxacin resistance, these agents can be used either in high dose monotherapy or rather in combination with other antibiotics, concerning the chance of rapid resistance development during monotherapy. The establishment of clinical breakpoints for agents other than SXT is strongly required in the near future. The most reliable antibiotic susceptibility testing method for alternative antibiotics should urgently be declared. Clinicians have to consider that *S. maltophilia* as a co-pathogen or co-colonizer in polymicrobial infections can have negative impact on the success rate of antibiotic treatment and clinical outcome.
